# An oxygen sensitive self-decision making engineered CAR T-cell

**DOI:** 10.1038/srep39833

**Published:** 2017-01-20

**Authors:** Alexandre Juillerat, Alan Marechal, Jean Marie Filhol, Yannick Valogne, Julien Valton, Aymeric Duclert, Philippe Duchateau, Laurent Poirot

**Affiliations:** 1 Cellectis Inc, 430E, 29th street, NYC, NY 10016, USA; 2 Cellectis, 8 rue de la croix Jarry, 75013, Paris

## Abstract

A key to the success of chimeric antigen receptor (CAR) T-cell based therapies greatly rely on the capacity to identify and target antigens with expression restrained to tumor cells. Here we present a strategy to generate CAR T-cells that are only effective locally (tumor tissue), potentially also increasing the choice of targetable antigens. By fusing an oxygen sensitive subdomain of HIF1α to a CAR scaffold, we generated CAR T-cells that are responsive to a hypoxic environment, a hallmark of certain tumors. Along with the development of oxygen-sensitive CAR T-cells, this work also provides a basic framework to use a multi-chain CAR as a platform to create the next generation of smarter self-decision making CAR T-cells.

Adoptive cell therapies using T cells presenting chimeric antigen receptors (CAR) at their surface have produced exciting anticancer activities over the past few years^1^,^2^,^3^. Although clinical studies have demonstrated the potential of this technology, they raised concerns associated with “on-target off-tumor” effect, particularly in healthy tissues with potential low expression of the targeted antigen^4^. The ability to control functional responses in T-cell based therapeutic strategies is thus a key issue. Therefore the development of technologies that allow improving the discrimination between healthy tissue and cancer cells would present extremely valuable advantages.

Synthetic biology applies many of the principles of engineering to the field of biology in order to create biological devices which can ultimately be integrated into increasingly complex systems. The ability to engineer synthetic systems in T-cells that are responding to multiple inputs would benefit adoptive immunotherapy using engineered T-cells^5^. The past years have seen the emergence of strategies to spatiotemporally control CAR T-cells, including those relying on the addition of exogenous small molecules or monoclonal antibodies to regulate^6^,^7^,^8^,^9^,^10^,^11^ or terminate^12^,^13^,^14^ CAR T cell functions. Alternatively, to achieve optimal tuning of CAR T-cell targeting and functional properties, researchers have developed novel approaches based on the use of combinatorial antigen targeting, such as trans-signaling CARs^15^,^16^,^17^, inhibitory CARs^18^, tandem CARs^19^ and synthetic Notch receptors^20^,^21^. Integration of endogenous environmental signals, in addition to antigen recognition, may represent a valuable advancement to improve the control of the CAR T-cell technology. An attractive strategy to discriminate between healthy tissue and cancer cells would be to rely on key peculiarities of the tumor microenvironment. Tumor microenvironment has been associated, *inter alia*, with depletion in nutrients, low extracellular pH (acidosis) and low oxygenation (hypoxia). In particular, hypoxic microenvironment is a hallmark of tumor microenvironment, with oxygenation levels often found below 1–2%^22^,^23^. Hypoxic microenvironment could thus represent an interesting feature to be exploited for the CAR-T cell technology to improve discrimination between tumoral and healthy tissues.

Here we report an approach that takes advantage of environmental signal integration within a CAR design. We developed a strategy in which the low oxygen concentration is used as an environmental signal of tumor microenvironment to allow manipulating the CAR T-cell response. In particular, we showed in an *in vitro* model, that the surface presentation of a multichain CAR fused to sub-domains of the hypoxia-inducible factors 1-alpha (HIF1α) as well as the cytolytic properties of such CAR T-cells can be modulated by variations in the oxygen level. Besides providing additional levels of safety by minimize “on-target/off-tumor” effects, integration of tumor “microenvironment sensors” may also expand the number of surface antigens available for therapeutic purposes.

## Results

### Design of an oxygen sensitive chimeric antigen receptor

**Figure 1:**
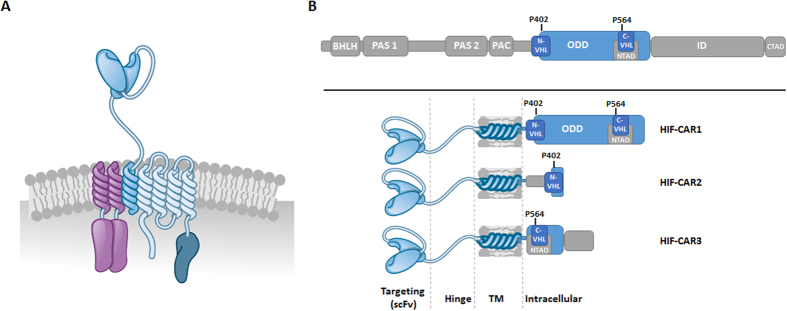
Schematic representations of the framework of an oxygen sensitive multichain CAR. (**A**) Organization of the engineered mcCAR based on FcεRI. (**B**) Design of the alpha chains that integrate an oxygen sensitive domain, leading to the three HIF-CARs. The following domains of HIF1α are shown: BHLH: basic helix-loop-helix domain, PAS: Per-Arnt-Sim homology domain, PAC: PAS-associated C-terminal domain, ODD: Oxygen-Dependent Degradation domain, N-TAD: N-terminal Transactivation Domain, C-TAD: C-terminal Transactivation Domain, N-VHL: N-terminal von Hippel–Lindau recognition site, ID: inhibitory domain and C-VHL: C-terminal von Hippel–Lindau recognition site. Reproduced with permission from Cellectis Group.

We aimed at engineering a T-cell that requires a double input, represented by the antigen recognition and the hypoxic tumor microenvironment, to obtain an optimal output, i.e. the T-cell activation and subsequent cytolytic properties. We further thought to develop a system where the two inputs will be “encoded” in a single chimeric antigen receptor (CAR) molecule without relying on additional transactivation of genes. To develop such CARs that could be self-controlled at the protein level by the oxygen concentration, we decided to engineer a recent modular CAR architecture that is based on the high affinity IgE receptor (FceRI)^24^, a scaffold we already used to control CAR-T cells using small molecules^6^. As previously reported, we engineered the gamma and beta chains of the FceRI protein to contain the immunoreceptor tyrosine-based activation motif (ITAM) from the z-chain of the CD3–T cell receptor (TCR) complex and the co-stimulatory domain of the tumor necrosis factor receptor superfamily member 9 (4-1BB, CD137) respectively (Fig. 1A). Finally, we engineered the alpha chain by replacing its native extracellular domain by a single-chain variable fragment (scFv) followed by a hinge domain derived from the T-cell surface glycoprotein CD8 alpha chain (CD8α).

Hypoxia-inducible factors (HIFs) are transcription factors that play important roles in cellular responses to variation in oxygen levels^25^. Under normal oxygen concentration, HIFs are constitutively degraded through polyubiquitination and subsequent proteasomal degradation. Under reduced oxygen tension, the proteins are stabilized, altogether resulting in transcriptional activation. Remarkably, it has been reported in cultured cell lines, an exponential induction of the HIF1α protein as oxygen concentration is reduced over the range from 20.0 to 0.5%^26^. Protein levels were however minimally impacted from 20% down to about 3% oxygen, with a massive increase in protein amounts observed below 2% oxygen (in range with reported oxygen concentration in tumor microenvironment^22^,^23^). We thus thought to use oxygen sensitive subdomains of the human HIF1α, excluding the transcription activation domains, to create a CAR that would be responsive (protein degradation) to oxygen variation (Fig. 1B). We further fused oxygen sensitive domains of HIF1α to the C-terminal end of the CAR alpha chain. This particular architecture presenting the advantage of having the new O_2_-sensor function directly integrated in the CAR scaffold.

In particular, we focused on three fragments of the HIF1α that contain key proline residues (P402 and P564) known to be hydroxylated in normoxia and involved in interactions with the von Hippel-Lindau tumor suppressor E3 ubiquitin ligase (VHLE3) multi-protein complex^27^,^28^,^29^. We thus designed and constructed three HIF-CARs by fusing the amino acids 380–603 (large domain, HIF-CAR1), 344–417 (Nter domain, HIF-CAR2) or 530–652 (Cter domain, HIF-CAR3) of the HIF1α (Fig. 1B) to the C-terminal end of the alpha chain via a glycine-serine (-GS-) linker. We focused on CARs targeting the well described CD19-antigen as a proof of concept^13^,^30^. We further relied on the vectorization of the CAR molecule via mRNA electroporation of primary T-cells as this allows sustainable expression of CARs for at least 48 h hours while permitting to efficiently control the stoichiometry (1 α/1 β/2 γ) of each chain of the oligomeric complex.

### Low oxygen concentration as an input: CAR surface expression

**Figure 2:**
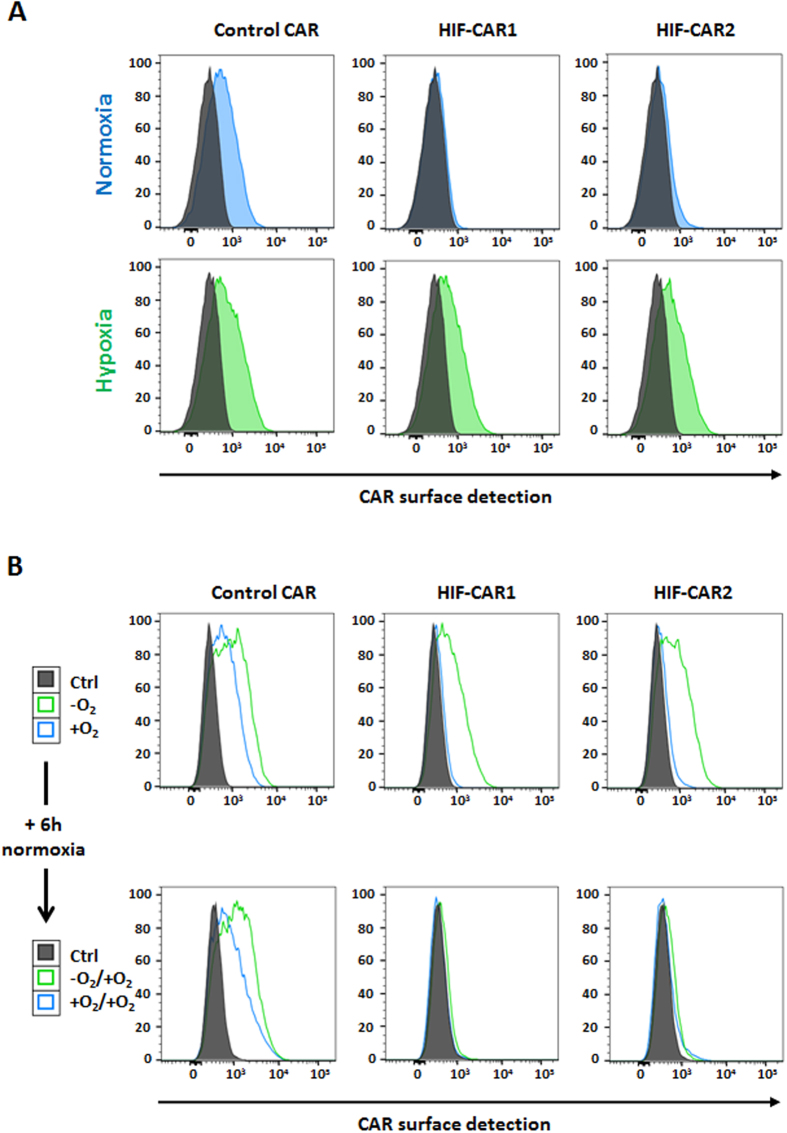
Impact of the oxygen level on the surface presentation of the engineered CAR. (**A**) Flow cytometry histogram representations of the detection of the different CARs (control, HIF-CAR1 and HIF-CAR2) under hypoxic or normoxic conditions. (**B**) Flow cytometry histogram representations of the switching-off property of the HIF-CARs upon removal of the hypoxia input. The detection of the F(ab’)2 region of the scFv is shown in representative experiments.

At first, to demonstrate that oxygen concentration can be used as an input, we focused on measuring the cell surface expression of the engineered CARs. To define the reachable ranges of surface expression of the CARs in hypoxia versus normoxia conditions, we performed a dose response experiment (doses of transfected mRNA) for the three HIF-CARs and a control CAR lacking the oxygen-sensitive domain. Activated primary T-cells transfected with the different CARs were incubated for 20 hours either in classical cell culture conditions or in an artificially created hypoxic environment. Based on previous reports on the biochemistry of the native HIF1α, we focused on two commonly used oxygen concentration: 21% for normoxia and below 1% for hypoxia^26^. We then monitored the surface presentation of the CARs by tracking the targeting scFv^13^,^30^. As expected, the variation in oxygen concentration only weakly influenced the surface detection of the control CAR (positive CAR T-cells: 51% in normoxia versus 66% in hypoxia, Fig. 2A and Supplementary Fig. 1). However, under hypoxic conditions the surface presentation of the HIF-CAR1 (positive CAR T-cells: 7% in normoxia versus 53% in hypoxia) and HIF-CAR2 (positive CAR T-cells: 11% in normoxia versus 58% in hypoxia) was markedly improved when compared to normoxia (Fig. 2A and Supplementary Fig. 1). In contrast, the surface expression of the CAR containing the Cter (HIF-CAR3) portion of HIF1α was only weakly controlled by the oxygen level (Supplementary Fig. 2). We then investigated if the expression of these HIF-CARs can be switched-off by removing the hypoxia input. T-cells transfected with the HIF-CAR1 and HIF-CAR2 from the previous hypoxic conditions were thus further incubated under normoxia conditions for an additional 6 hours. The surface expression of the CARs was then compared to CAR T-cells that were only incubated in normoxia. Importantly, the surface expression of the two HIF-CAR T-cells originally cultured under hypoxic conditions was markedly reduced (positive CAR T-cells: 9% for HIF-CAR1 and 14% for HIF-CAR2), confirming the reactivity (switching off) of the engineered CAR to variation in oxygen levels (Fig. 2B), a feature not observed for the control CAR (positive CAR T-cells: 66%).

### HIF-CAR are rapidly switched-down after removal of inducing signal (hypoxia)

One key feature of such reactive switch systems is their ability to quickly return to their off state in the absence of the inducing signal (hypoxia). In particular, this characteristic would be of prime interest to protect distant healthy tissues from off-tumor/on-target effects. The integration within the CAR of a hypoxia-sensing domain that is functional at the protein level is expected to more rapidly lead to a switching-off, opposite to approaches relaying on controlling gene transactivation.

**Figure 3:**
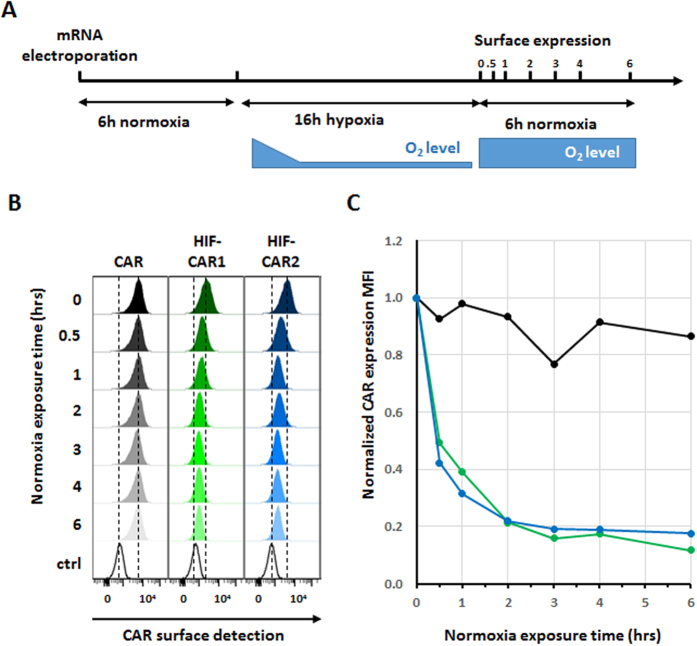
Determination of the CAR surface presentation decay after removal of the hypoxia input. (**A**) Schematic representation of the experimental setup. (**B**) Flow cytometry histogram representation of the CAR detection at the surface of primary T-cell after removal of the hypoxia input over a 6 hours period. The detection of the F(ab’)2 region of the scFv is shown. (**C**) Time course analysis of CARs surface presentation decay after removal of the hypoxia input (Control CAR in black, HIF-CAR1 in Green and HIF-CAR2 in blue). The MFI values were normalized to 1 for each CAR at the time at which the normoxia condition were reestablished.

Therefore, we further characterized the decay dynamics of the different CARs after removal of the inducing signal (hypoxia, Fig. 3A). After a 6 hours recovery in normoxia directly following the mRNA electroporation, the different CAR T-cells were cultures in hypoxia for 16 h to maximize their surface presentation. Engineered T-cells were then transferred in normoxic conditions and we monitored the surface presentation decay over a 6 hour period (Fig. 3B), a total of less than 30 hours post electroporation perfectly compatible with the mRNA vectorization. We then determined that the CAR surface expression was decreased by 80% in approximately 2 hours for the HIF-CAR1 and HIF-CAR2, considering the mean fluorescence intensity (Fig. 3C) and that this residual CAR expression remained stable up to 6 hours. As expected from the previous experiments, the cell surface expressions of the HIF-CAR3 and control CAR were unaffected by the removal of the hypoxia stimulus (Supplementary Fig. 3).

### Combinatorial input signal is required for enhanced cytolytic properties

Having shown that a robust surface presentation of the HIF-CAR depended on a low oxygen concentration, we next intended to demonstrate that this concept of dual inputs signals circuit (low oxygen concentration and targeted antigen) can lead to an active output, i.e. T-cell cytolytic functions. Therefore, we compared in an *in vitro* system, the cytolytic properties of T-cells transfected with the control CAR or its oxygen sensitive counterpart cultured under hypoxic or normoxic conditions. We further focused on the HIF-CAR that contains the longer domain of HIF1α.

**Figure 4:**
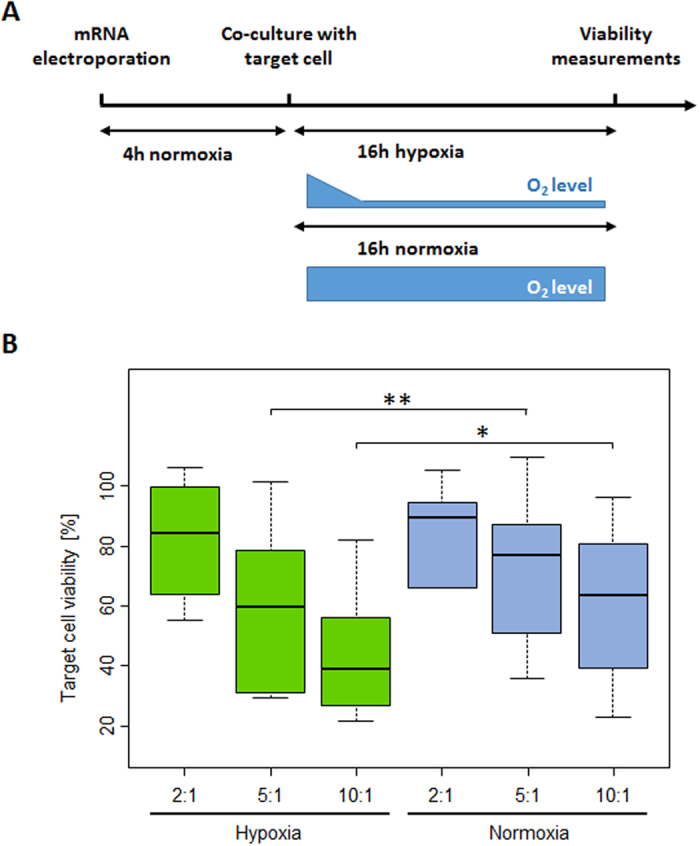
Cytolytic properties of the engineered HIF-CAR T-cells. (**A**) Schematic representation of the experimental setup. (**B**) The effect of the difference of oxygen levels (normoxia and hypoxia) on the cytolytic capacities of the of the CAR T cells toward model antigen presenting cell was assessed in a luciferase-based assay. Boxplots representing the percentage of viable Daudi target cell after coculture with engineered HIF-CAR1 T-cells. E/T = 2, E/T = 5, E/T = 10. E/T denote the effector/target ratios, N = 6 with each experiments done in three technical replicates. Significant differences in viability between normoxic and hypoxic conditions are indicated. Significance is determine by a standard paired t-test, *p ≤ 0.05,**p ≤ 0.01.

Following the mRNA electroporation and a 4 hours recovery, CAR T-cells were coincubated, at various ratio, for 16 h with Daudi target cells under either normoxic or hypoxic conditions (Fig. 4A). At the end of the co-culture, we calculated the difference in viability of the target cells in function of the oxygen condition and the ratio of effector and target cells (E/T) (Fig. 4B and Supplementary Fig. 4). We first confirmed that the cytolytic properties of the HIF engineered CAR T-cells were not completely abrogated in the normoxic condition (Fig. 4B). This was expected as we showed (Fig. 3C) that despite being very rapidly switched-down, a basal level of CAR surface presentation remained detectable in normoxia (representing less than 20% of the maximum). However we found that the ability of the engineered HIF-CAR T-cells to kill target cells were significantly improved (p-values =0.003 for the 5 E/T ratio and 0.0276 for the 10 E/T ratio) in hypoxia versus normoxia (Fig. 4B). In contrast, for co-cultures with the control CAR T-cell, we did not observed a significant improvement of the cytolytic properties under hypoxic conditions versus normoxic conditions (at the 2:1 E/T ratio, target cell killing was slightly but significantly more important in normoxia versus hypoxia, Supplementary Fig. 4).

Altogether, the results presented here provide the proof of principle of engineering a CAR scaffold to create an integrated oxygen-based self-decision making T-cell, that allows tuning the cytolytic properties of CAR T-cells depending on the microenvironment. Beyond these first steps, additional studies will be necessary to fully assess the therapeutic potential of this technology. In particular, mastering the stable integration of these new multichain CAR architectures in the genome of primary T-cells, will open novel possibilities for further *in vitro* and *in vivo* characterizations. Stable expression of the HIF-CAR would notably allow to complete the biochemical characterization of these engineered receptors, by obtaining oxygen response curves to determine thresholds of oxygen concentrations where the system could act as a switch on/off or as a rheostat. Additional engineering of the fusion of the alpha chain and the HIF1α sub-domains (or potentially other oxygen sensitive domains) could also open the possibility to further modulate the response to oxygen variation. Finally, because of its potential to enhance the safety of CAR T-cell immunotherapies, *in vivo* studies will be required to assess fundamental CAR T-cells properties such as engraftments, proliferation, cytokine production and tumor control.

## Discussion

The recent years have seen the emergence of novel cancer immunotherapy approaches notably through the possibility to endow T cells with chimeric antigen receptors (CAR)^31^,^32^,^33^. However, the development of highly potent engineered T-cells requires the identification of target antigens that present very narrows expression profiles among all tissues with a high preference for those that are uniquely present on tumor cells. However, known non-life threatening off-tumor targeting may be tolerated) as clinical trial pointed out that “on-target off-tumor” may potentially leads to fatal issues^4^.

Parallel to the development of the CAR itself through optimizing the cytolytic activity or proliferative capacities of the engineered T-cells, several approaches were dedicated to improve the discrimination between cancer and healthy tissues using multiple engineered receptors. Such approaches mainly relied on expressing a CAR with two targeting moieties^19^ or expressing two CARs (targeting distinct antigens) in the same T-cell. This has been achieved by splitting the activation and costimulation on the two receptors^15^,^16^ (logic AND gate) or by combining activating and inhibiting CARs (logic NOT gate)^18^. More recently, Lim and coworker improved the AND gate combinatorial detection of multiple antigens strategy by implementing an engineered Notch-based receptors that will function independently from the TCR pathway activated by the CAR^20^,^21^.

However, a supposed disadvantage of any combinatorial antigen targeting approaches resides in the possible target antigen modulation leading to tumor escape, with increasing probability of escape with the requirement of additional antigen targeting. Altogether, the decrease of the targeted antigen expression could potentially render the engineered CAR system either partially or completely unresponsive (AND gates) or non-protective (NOT gates)^34^. In addition, the selection of combination of suitable target antigens may be difficult due to differential expression levels, thus requiring fine parallel optimization of the properties (expression levels, affinities) of the different CARs^18^.

Alternatively to these strategies that allow a spatial control of CAR T-cells, methods that enable potential spatio-temporal control the fate of CAR T-cells using exogenous small molecules or cytokines were also described^6^,^7^,^35^. In particular, Vera and colleagues have demonstrated the possibility to engineer a T-cell by taking advantage of the tumor microenvironment. They reverted the inhibitory effect of the IL4 cytokine by creating a double input system (target antigen and IL4) that promoted selective expansion of transgenic T cells only in the tumor microenvironment^35^.

In this report, we attempted to design a multi-input approach using single antigen targeting associated with an endogenous tumor environmental signal (hypoxia), altogether creating locally effective CAR T-cells. Toward this goal we designed a CAR that is sub-optimally presented at the surface of the T-cell under normal oxygen concentration. We further demonstrated that an increased surface expression along with improved cytolytic properties can be obtained under hypoxic conditions. It has been previously reported that oxygen concentrations in human solid tumors are highly heterogeneous and that approximately 50% of these solid tumors contain hypoxic tissues^22^,^23^. We believed that a basal level of CAR expression will be beneficial, as the CAR-T-cells have to recognize the target antigen to induce their activation and expansion properties; a complete absence of CAR expression would prevent this in the normoxic region of the tumor. Moreover, the resulting suboptimal CAR T-cells cytolytic properties would be boosted in the hypoxic tumor tissues. We hypothesized that the combination of the residual CAR expression in normoxia associated with low antigen expression in healthy tissue should not lead to deleterious cytotoxicity. However, further additional *in vitro* and more likely *in vivo* studies will be required to fully address this point. Furthermore, additional layers of control of the cytolytic properties of the HIF-CAR could be implemented. For example, it has been demonstrated that tuning the affinity of the targeting moiety (scFv) can also improve discrimination between tissues expressing different levels of target antigen^36^,^37^. Such particular feature would be of prime interest to minimize potential adverse events but also to widen the panel of possible targeted antigens. Importantly, we also showed that the oxygen CAR-integrated microenvironment sensors are prone to rapid switch-down, which would protect distant healthy tissues. In addition to our first proof of concept of a stand-alone use of the engineered oxygen sensitive CAR, one can imagine combining this strategy with multi-receptor AND and NOT gates to further enhance the control of the engineered cells. While there is still much development ahead, we envisioned that these approaches will contribute to program therapeutic cells with improved efficiency and safety profiles.

## Methods

All individual chains of the CAR architecture were amplified by PCR using oligo pairs α-chain-F/ α-chain-R, α-chain-F/ α-chain-HIF-R1, α-chain-F/ α-chain-HIF-R2, β-chain-F/ β-chain-R and γ-chain-F/ γ-chain-R prior to mRNA synthesis (Supplementary Table 2). mRNA encoding the α-chain, β-chain or γ-chain were *in vitro* transcribed from the PCR product and polyadenylated using the mMessage mMachine T7 Ultra kit (Life technologies) following the manufacturer’s instructions. RNAs were purified with RNeasy columns (Qiagen), eluted in cytoporation medium T and quantified by measuring absorbance at 260 nm using a Nanodrop ND-1000 spectrophotometer. Quality of the RNA was verified on a denaturing formaldehyde/MOPS agarose gel.

### Transfection

T lymphocytes were transfected by electrotransfer of messenger RNA using an AgilePulse MAX system (Harvard Apparatus) 4 to 6 days after activation. Following removal of activation beads, cells were pelleted, resuspended in cytoporation medium T at 28 × 10^6^ cells/ml. 5 × 10^6^ cells were mixed with the previously synthetized mRNA into a 0.4 cm cuvette. This particular setup allowed maintaining an identical stoichiometry (1 α/1 β/2 γ) of each chain for all constructs.

The electroporation consisted of two 0.1 ms pulses at 1200 V followed by four 0.2 ms pulses at 130 V. Following electroporation, cells were diluted into 2 mL culture medium and cultured in a humidified 37 °C/5% CO_2_ incubator for 4 hours. The cells were then transferred in 96 well plate and incubated either at 37 °C/ 5% CO_2_ (referred as normoxia) or at 37 °C with low O_2_ concentration (referred as hypoxia) for 16 h. Hypoxic conditions were created using an atmosphere generation system (2.5 L AnaeroJAR assembly, Anaerogen 2.5 L, Anaerobic indicator BR0055 Oxoid). According to the manufacturer, the level of oxygen is decreased below 1% in less than 30 minutes. A fraction of the cells from the hypoxia condition were kept and incubated after careful mixing at 37 °C/ 5% CO_2_ (normoxia) for 4–6 h.

### Flow cytometry

Primary labelling for the detection of the targeting α-chain was performed with anti-F(ab’)2-Biotin (goat anti-mouse IgG, F(ab’)2 fragment specific, Jackson Immunoresearch) in PBS FBS 2%, EDTA 2 mM, azide 0.1% for 20 min at 4 °C followed by a two washing steps with PBS FBS 2% EDTA 2 mM azide 0.1%. Secondary labelling was performed with Streptavidin-V450 (BD Pharmingen) in PBS FBS2% EDTA 2 mM azide 0.1% for 20 min at 4 °C followed by a washing step in PBS FBS2% EDTA 2 mM azide 0.1% and a washing step in PBS.

Following the extracellular labelling, the cell viability was monitored using efluor450 or efluor780 dyes (ebioscience) in PBS for 20 min 4 °C, followed by a washing step with PBS FBS2% EDTA 2 mM azide 0.1% and fixed in PFA 2%. Flow cytometry was performed using the MACSQUANT (Miltenyi Biotec) and data analysis was performed with the FlowJo software.

### Cytotoxicity assay

The cytolytic activity of engineered T-cells endowed with the different CARs was assessed using a Luciferase-based cytotoxicity assay. The target cells presenting the CAR target antigen (Daudi) stately expressed a Firefly Luciferase. 4 hours after mRNA electroporation the target cell populations was co-incubate in 96 well plate either at 37 °C/ 5% CO_2_ (referred as normoxia) or at 37 °C with low O_2_ concentration with various ratio of engineered effector CAR T cells (Effector/Target ratio of 2:1, 5:1 and 10:1) in a final volume of X-Vivo-15 media of 100 μL, for an overnight incubation. Viability of the CAR target antigen cell was assessed by quantifying the luciferase signal using ONE-Glo™ Luciferase Assay System (Promega) and analyzed with VICTOR Plate Reader.

### Statistical analysis

To evaluate the significance of the differences of Daudi cell lysis by CAR T cells between hypoxic and normoxic conditions, a standard paired t-test was used on the different transfections.

## Additional Information

**How to cite this article:** Juillerat, A. *et al*. An oxygen sensitive self-decision making engineered CAR T-cell. *Sci. Rep.* **7**, 39833; doi: 10.1038/srep39833 (2017).

**Publisher's note:** Springer Nature remains neutral with regard to jurisdictional claims in published maps and institutional affiliations.

## Supplementary Material

Supplementary Information

## References

[b1] Brentjens R. J. . CD19-targeted T cells rapidly induce molecular remissions in adults with chemotherapy-refractory acute lymphoblastic leukemia. Sci Transl Med 5, 177ra138 (2013). 10.1126/scitranslmed.3005930 PMC3742551 23515080

[b2] Grupp S. A. . Chimeric antigen receptor-modified T cells for acute lymphoid leukemia. N Engl J Med 368, 1509–1518 (2013). 23527958 10.1056/NEJMoa1215134 PMC4058440

[b3] Kalos M. . T cells with chimeric antigen receptors have potent antitumor effects and can establish memory in patients with advanced leukemia. Sci Transl Med 3, 95ra73 (2011). 10.1126/scitranslmed.3002842 PMC3393096 21832238

[b4] Morgan R. A. . Case report of a serious adverse event following the administration of T cells transduced with a chimeric antigen receptor recognizing ERBB2. Mol Ther 18, 843–851 (2010). 20179677 10.1038/mt.2010.24 PMC2862534

[b5] Chakravarti D. & Wong W. W. Synthetic biology in cell-based cancer immunotherapy. Trends Biotechnol 33, 449–461 (2015). 26088008 10.1016/j.tibtech.2015.05.001 PMC4509852

[b6] Juillerat A. . Design of chimeric antigen receptors with integrated controllable transient functions. Sci Rep 6, 18950 (2016). 26750734 10.1038/srep18950 PMC4707440

[b7] Wu C. Y., Roybal K. T., Puchner E. M., Onuffer J. & Lim W. A. Remote control of therapeutic T cells through a small molecule-gated chimeric receptor. Science 350, aab4077 (2015). 26405231 10.1126/science.aab4077 PMC4721629

[b8] Ma J. S. . Versatile strategy for controlling the specificity and activity of engineered T cells. Proc Natl Acad Sci USA 113, E450–458 (2016). 26759368 10.1073/pnas.1524193113 PMC4743826

[b9] Rodgers D. T. . Switch-mediated activation and retargeting of CAR-T cells for B-cell malignancies. Proc Natl Acad Sci USA 113, E459–468 (2016). 26759369 10.1073/pnas.1524155113 PMC4743815

[b10] Tamada K. . Redirecting gene-modified T cells toward various cancer types using tagged antibodies. Clin Cancer Res 18, 6436–6445 (2012). 23032741 10.1158/1078-0432.CCR-12-1449

[b11] Urbanska K. . A universal strategy for adoptive immunotherapy of cancer through use of a novel T-cell antigen receptor. Cancer Res 72, 1844–1852 (2012). 22315351 10.1158/0008-5472.CAN-11-3890 PMC3319867

[b12] Marin V. . Comparison of different suicide-gene strategies for the safety improvement of genetically manipulated T cells. Hum Gene Ther Methods 23, 376–386 (2012). 23186165 10.1089/hgtb.2012.050 PMC4015080

[b13] Poirot L. . Multiplex Genome-Edited T-cell Manufacturing Platform for “Off-the-Shelf” Adoptive T-cell Immunotherapies. Cancer Res 75, 3853–3864 (2015). 26183927 10.1158/0008-5472.CAN-14-3321

[b14] Straathof K. C. . An inducible caspase 9 safety switch for T-cell therapy. Blood 105, 4247–4254 (2005). 15728125 10.1182/blood-2004-11-4564 PMC1895037

[b15] Duong C. P., Westwood J. A., Berry L. J., Darcy P. K. & Kershaw M. H. Enhancing the specificity of T-cell cultures for adoptive immunotherapy of cancer. Immunotherapy 3, 33–48 (2011). 21174556 10.2217/imt.10.81

[b16] Wilkie S. . Dual targeting of ErbB2 and MUC1 in breast cancer using chimeric antigen receptors engineered to provide complementary signaling. J Clin Immunol 32, 1059–1070 (2012). 22526592 10.1007/s10875-012-9689-9

[b17] Krause A. . Antigen-dependent CD28 signaling selectively enhances survival and proliferation in genetically modified activated human primary T lymphocytes. J Exp Med 188, 619–626 (1998). 9705944 10.1084/jem.188.4.619 PMC2213361

[b18] Fedorov V. D., Themeli M. & Sadelain M. PD-1- and CTLA-4-based inhibitory chimeric antigen receptors (iCARs) divert off-target immunotherapy responses. Sci Transl Med 5, 215ra172 (2013). 10.1126/scitranslmed.3006597 PMC4238416 24337479

[b19] Grada Z. . TanCAR: A Novel Bispecific Chimeric Antigen Receptor for Cancer Immunotherapy. Mol Ther Nucleic Acids 2, e105 (2013). 23839099 10.1038/mtna.2013.32 PMC3731887

[b20] Morsut L. . Engineering Customized Cell Sensing and Response Behaviors Using Synthetic Notch Receptors. Cell 164, 780–791 (2016). 26830878 10.1016/j.cell.2016.01.012 PMC4752866

[b21] Roybal K. T. . Precision Tumor Recognition by T Cells With Combinatorial Antigen-Sensing Circuits. Cell 164, 770–779 (2016). 26830879 10.1016/j.cell.2016.01.011 PMC4752902

[b22] Brown J. M. & Wilson W. R. Exploiting tumour hypoxia in cancer treatment. Nat Rev Cancer 4, 437–447 (2004). 15170446 10.1038/nrc1367

[b23] Vaupel P. & Mayer A. Hypoxia in cancer: significance and impact on clinical outcome. Cancer Metastasis Rev 26, 225–239 (2007). 17440684 10.1007/s10555-007-9055-1

[b24] Kinet J. P. The high-affinity IgE receptor (Fc epsilon RI): from physiology to pathology. Annu Rev Immunol 17, 931–972 (1999). 10358778 10.1146/annurev.immunol.17.1.931

[b25] Semenza G. L. Hypoxia-inducible factors: mediators of cancer progression and targets for cancer therapy. Trends Pharmacol Sci 33, 207–214 (2012). 22398146 10.1016/j.tips.2012.01.005 PMC3437546

[b26] Jiang B. H., Semenza G. L., Bauer C. & Marti H. H. Hypoxia-inducible factor 1 levels vary exponentially over a physiologically relevant range of O2 tension. Am J Physiol 271, C1172–1180 (1996). 8897823 10.1152/ajpcell.1996.271.4.C1172

[b27] Epstein A. C. . C. elegans EGL-9 and mammalian homologs define a family of dioxygenases that regulate HIF by prolyl hydroxylation. Cell 107, 43–54 (2001). 11595184 10.1016/s0092-8674(01)00507-4

[b28] Masson N., Willam C., Maxwell P. H., Pugh C. W. & Ratcliffe P. J. Independent function of two destruction domains in hypoxia-inducible factor-alpha chains activated by prolyl hydroxylation. EMBO J 20, 5197–5206 (2001). 11566883 10.1093/emboj/20.18.5197 PMC125617

[b29] Paltoglou S. & Roberts B. J. HIF-1alpha and EPAS ubiquitination mediated by the VHL tumour suppressor involves flexibility in the ubiquitination mechanism, similar to other RING E3 ligases. Oncogene 26, 604–609 (2007). 16862177 10.1038/sj.onc.1209818

[b30] Kugler M. . Stabilization and humanization of a single-chain Fv antibody fragment specific for human lymphocyte antigen CD19 by designed point mutations and CDR-grafting onto a human framework. Protein Eng Des Sel 22, 135–147 (2009). 19188138 10.1093/protein/gzn079

[b31] Abate-Daga D. & Davila M. L. CAR models: next-generation CAR modifications for enhanced T-cell function. Mol Ther Oncolytics 3, 16014 (2016). 27231717 10.1038/mto.2016.14 PMC4871190

[b32] Sadelain M., Brentjens R. & Riviere I. The basic principles of chimeric antigen receptor design. Cancer Discov 3, 388–398 (2013). 23550147 10.1158/2159-8290.CD-12-0548 PMC3667586

[b33] van der Stegen S. J., Hamieh M. & Sadelain M. The pharmacology of second-generation chimeric antigen receptors. Nat Rev Drug Discov 14, 499–509 (2015). 26129802 10.1038/nrd4597 PMC6410718

[b34] Anurathapan U. . Kinetics of tumor destruction by chimeric antigen receptor-modified T cells. Mol Ther 22, 623–633 (2014). 24213558 10.1038/mt.2013.262 PMC3945803

[b35] Leen A. M. . Reversal of tumor immune inhibition using a chimeric cytokine receptor. Mol Ther 22, 1211–1220 (2014). 24732709 10.1038/mt.2014.47 PMC4048899

[b36] Caruso H. G. . Tuning Sensitivity of CAR to EGFR Density Limits Recognition of Normal Tissue While Maintaining Potent Antitumor Activity. Cancer Res 75, 3505–3518 (2015). 26330164 10.1158/0008-5472.CAN-15-0139 PMC4624228

[b37] Liu X. . Affinity-Tuned ErbB2 or EGFR Chimeric Antigen Receptor T Cells Exhibit an Increased Therapeutic Index against Tumors in Mice. Cancer Res 75, 3596–3607 (2015). 26330166 10.1158/0008-5472.CAN-15-0159 PMC4560113

