# Supplementary material for: An oxygen sensitive self-decision making engineered CAR T-cell
Source: Sci Rep. 2017 Jan 20;7:39833. doi: 10.1038/srep39833 (PMC5247770; doi:10.1038/srep39833)
Supplement: Supplementary Information [file srep39833-s1.pdf]

Supplementary information for:

An oxygen sensitive self-decision making engineered CAR T-cell

Alexandre Juillerat[1], Alan Marechal[2], Jean Marie Filhol[2], Yannick Valogne[2], Julien Valton[1], Aymeric Duclert[2] Philippe Duchateau[2] and Laurent Poirot[2]

[1]Cellectis Inc, 430E, 29th street, NYC, NY 10016, USA

[2]Cellectis, 8 rue de la croix Jarry, 75013 Paris

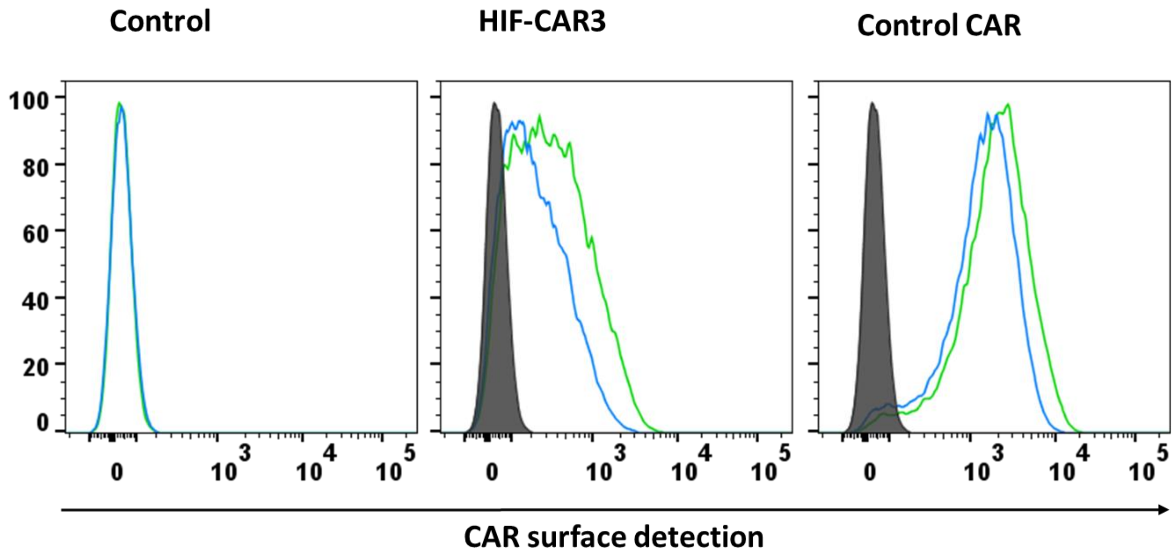

Supplementary figure 1: Impact of the oxygen level on the surface presentation of the engineered HIF-CAR3. FACS histogram representations of the detection of the different CARs (control and HIF-CAR3) under hypoxic or normoxic conditions.

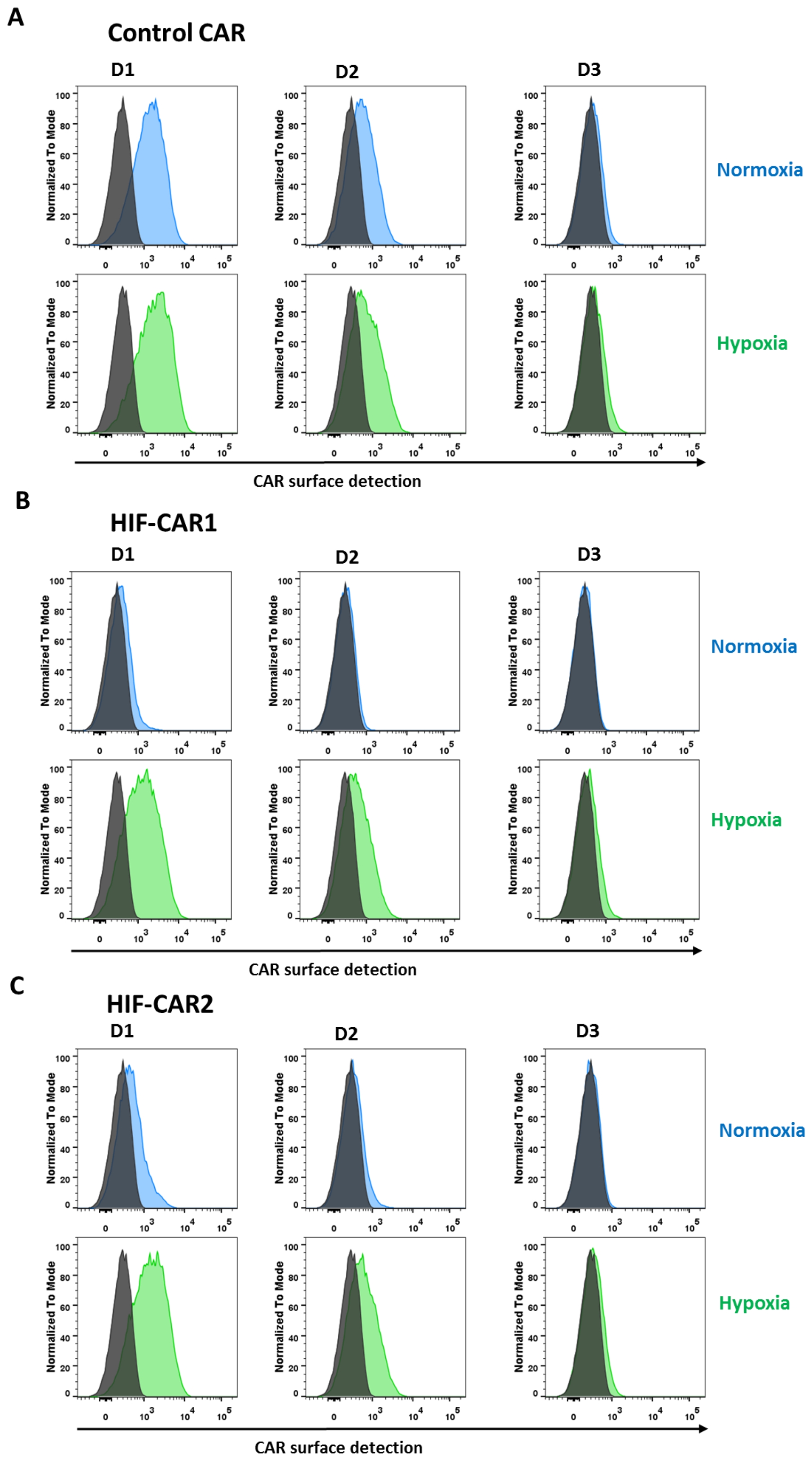

Supplementary figure 2: Impact of the oxygen level on the surface presentation of the engineered CAR. FACS histogram representations of the detection of the different CARs (control, HIF-CAR1 and HIF-CAR2) under hypoxic or normoxic conditions with different doses of transfected mRNA.

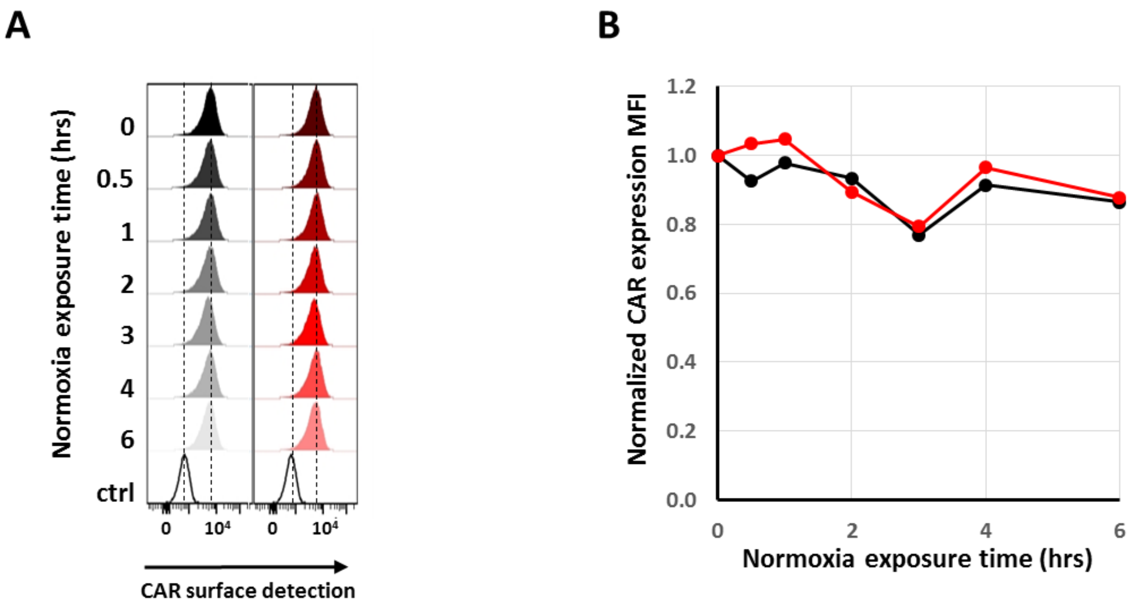

Supplementary figure 3: Determination of the CAR surface presentation decay after removal of the hypoxia input. (a) FACS histogram representation of the CAR detection (HIF-CAR3 in red and control CAR in black) at the surface of primary T-cell after removal of the hypoxia input over a 6h period. The detection of the Fab'2 region of the scFv is shown. (b) Time course analysis of CARs (HIF-CAR3 in red and control CAR in black) surface presentation decay after removal of the hypoxia input. The MFI values were normalized to 1 for each CAR at the time at which the normoxic condition were re-established.

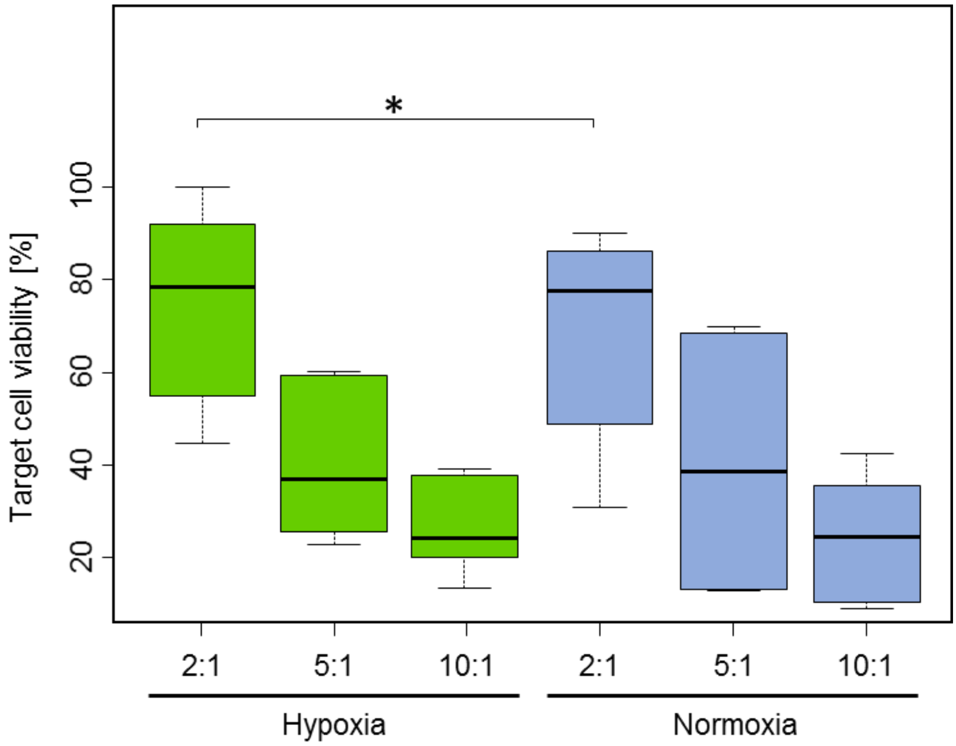

Supplementary figure 4: Cytolytic properties of the control CAR T-cells. The effect of the difference of oxygen levels (normoxia and hypoxia) on the cytolytic capacities of the of the CAR T cells toward model antigen presenting cell was assessed in a luciferase-based assay. Boxplots representing the percentage of viable Daudi target cell after coculture with CAR T-cells. E/T=2, E/T=5, E/T=10. E/T denote the effector/target ratios, N=6 with each experiments done in three technical replicates. Significant differences in viability between normoxic and hypoxic conditions are indicated. Significance is determine by a standard paired t-test, * = $p \leq 0.05$.
